# Supplementary material for: Biohybrid Microrobots Based on Jellyfish Stinging Capsules and Janus Particles for In Vitro Deep‐Tissue Drug Penetration
Source: Small Sci. 2025 Feb 11;5(6):2400551. doi: 10.1002/smsc.202400551 (PMC12168594; doi:10.1002/smsc.202400551)
Supplement: Supplementary file 1 — Supplementary Material [file SMSC-5-2400551-s001.zip › smsc202400551-sup-0001-SuppData-S1.pdf]

Supplementary information

Biohybrid Microrobots Based on Jellyfish Stinging Capsules and Janus Particles for In Vitro Deep-Tissue Drug Penetration

Sinwook Park[1, 3], Noga Barak[2], Tamar Lotan[2], and Gilad Yossifon[1, 3*]

[1]School of Mechanical Engineering, Tel-Aviv University, Tel Aviv, 6997801, Israel

[2]Marine Biology Department, The Leon H. Charney School of Marine Sciences, University of Haifa, Haifa, 310330, Israel

[3]Department of Biomedical Engineering, Tel-Aviv University, Tel Aviv, 6997801, Israel

* Corresponding author: gyossifon@tauex.tau.ac.il

Movie S1: Two movies show the transport of a biohybrid microbot carrying jellyfish capsules: one driven by a unified external AC electric field (2 kHz, $15V_{pp}$) within 0.1 mM NaCl without a magnetic field, and the other by a combined external rotating magnetic field (rotation speed with 100 rpm) and an AC electric field (2 MHz, $15V_{pp}$) within 10 mM NaCl, corresponding to Figs. 2a and 2b, respectively.

Movie S2: Self-propulsion behavior of JPs in different concentrations of NaCl solution under the application of an electric field with varying frequencies (2 kHz, 10 kHz, 50 kHz, and 2 MHz) and magnetic rolling.

Movie S3: Combined two movies showing the transport and activation of the loaded jellyfish capsules by enzyme triggering, focused on biohybrid microrobots with various configuration of JPs corresponding to Fig. 3a.

Movie S4: Transport and molecule ejection of Toluidine Blue O, (TBO) in 10mM NaCl solution corresponding to Fig. 3b.

Movie S5: Transport and molecule ejection of Acridine Orange in 10mM NaCl solution corresponding to Fig. 3c.

Movie S6: Transport of the microbots towards the target spheroid using magnetic rolling assisted with electric field corresponding Fig. 4b and representative event of molecule injection of Toluidine Blue O, TBO deep into the spheroid corresponding to Fig. 4c.

Movie S7: Molecule injection of Acridine Orange hemi(zinc chloride) salt deep into the spheroid from the loaded jellyfish capsules of the biohybrid microrobot by enzyme triggering corresponding to Fig. 4d.

Movie S8: Transport of biohybrid microbots toward swimming healthy C. elegans and their unsuccessful attempts to penetrate capsule tubules into the target worms due to the latter's fast mobility.

Movie S9: The penetration of the biohybrid microbots' tubules from the small and large capsule into paralyzed *C. elegans* by enzyme triggering corresponding to Figs. 5b and c, respectively.

Movie S10: Combined movies of control tests to observe the reactions of C. elegans under various potential stimuli: a) Control test with only enzyme introduction without an external electric field. b) Control test introducing both the enzyme and applying an external electric field with magnetic rolling, using paralyzed worms. c) Control test without the penetration of tubules under conditions of electric/magnetic fields and enzyme introduction. d) Test showing the penetration of tubules from the small capsule into non-paralyzed C. elegans by enzyme triggering.

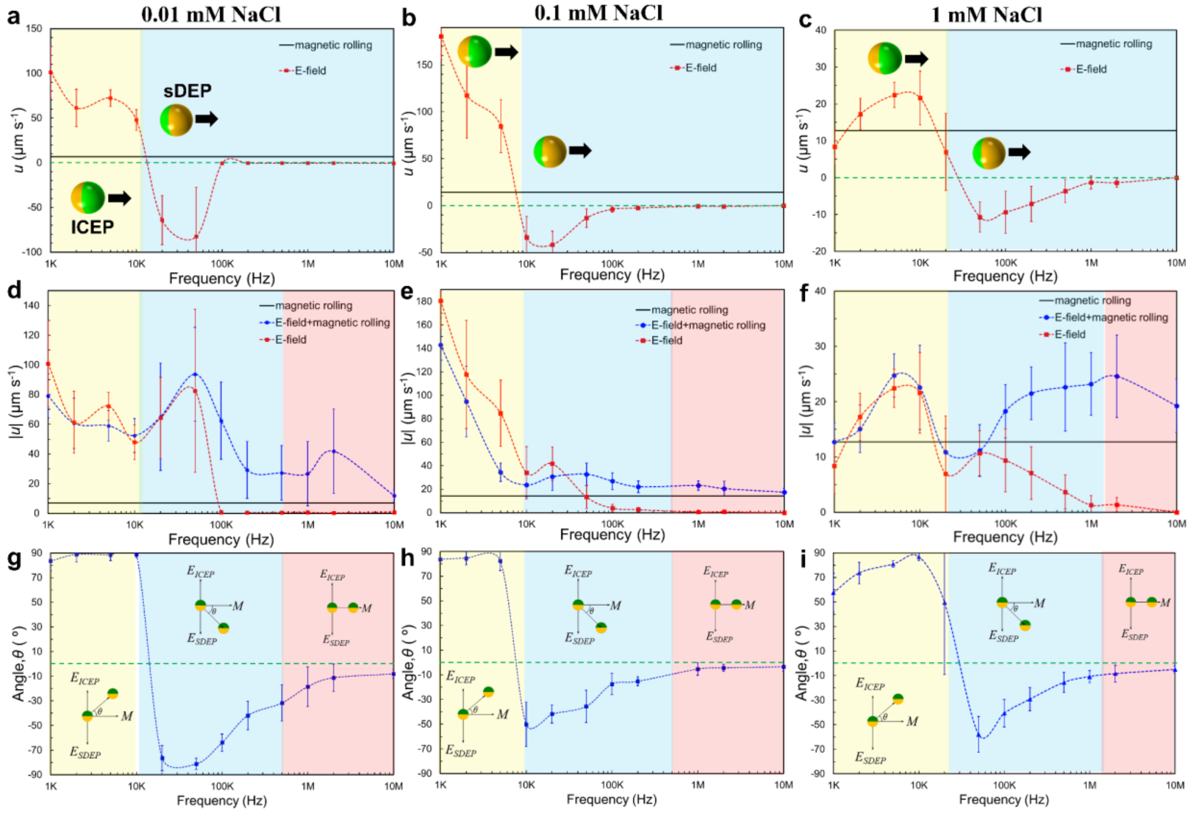

Figure S1. Propulsion behavior of Janus particles in different concentrations of NaCl solution under the application of an electric field and magnetic rolling. (a, b, c) Velocity profiles of Janus particle self-propulsion under decoupled magnetic rolling and electric field application within NaCl concentrations of 0.01, 0.1, and 1 mM, respectively. (d, e, f) Absolute velocity magnitudes under three conditions: only magnetic rolling, coupled magnetic rolling and electric field, and only electric field, within NaCl concentrations of 0.01, 0.1, and 1 mM, respectively. (Refer to representative movie S2 as an example of Janus particle movement.) (g, h, i) Changes in the angle (θ) of the direction of motion relative to the direction of only magnetic rolling (set as the horizontal axis) when both the electric field at various frequencies and magnetic rolling are coupled. Yellow, green, and red rectangles indicate dominance of ICEP, sDEP, and magnetic rolling transport of Janus particles, respectively, upon the applied frequency of the electric field.

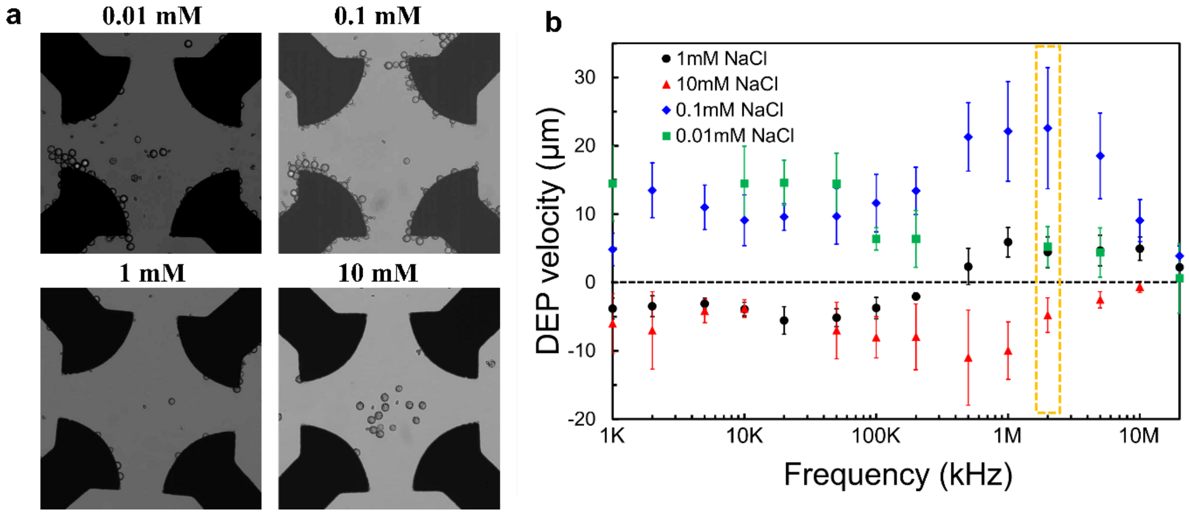

Figure S2. DEP characterization of the jellyfish capsules under different concentrations of NaCl solution. (a) Microscopic images illustrating pDEP and nDEP behaviors of the capsules at NaCl concentrations of 0.01, 0.1, and 1 mM, and 10 mM NaCl, under an applied electric field (2 MHz, $10V_{pp}$). (b) Experimental DEP spectra of jellyfish capsules in various NaCl concentrations. The orange dashed rectangle highlights distinct DEP behaviors of jellyfish capsules at 2 MHz corresponding to part (a).

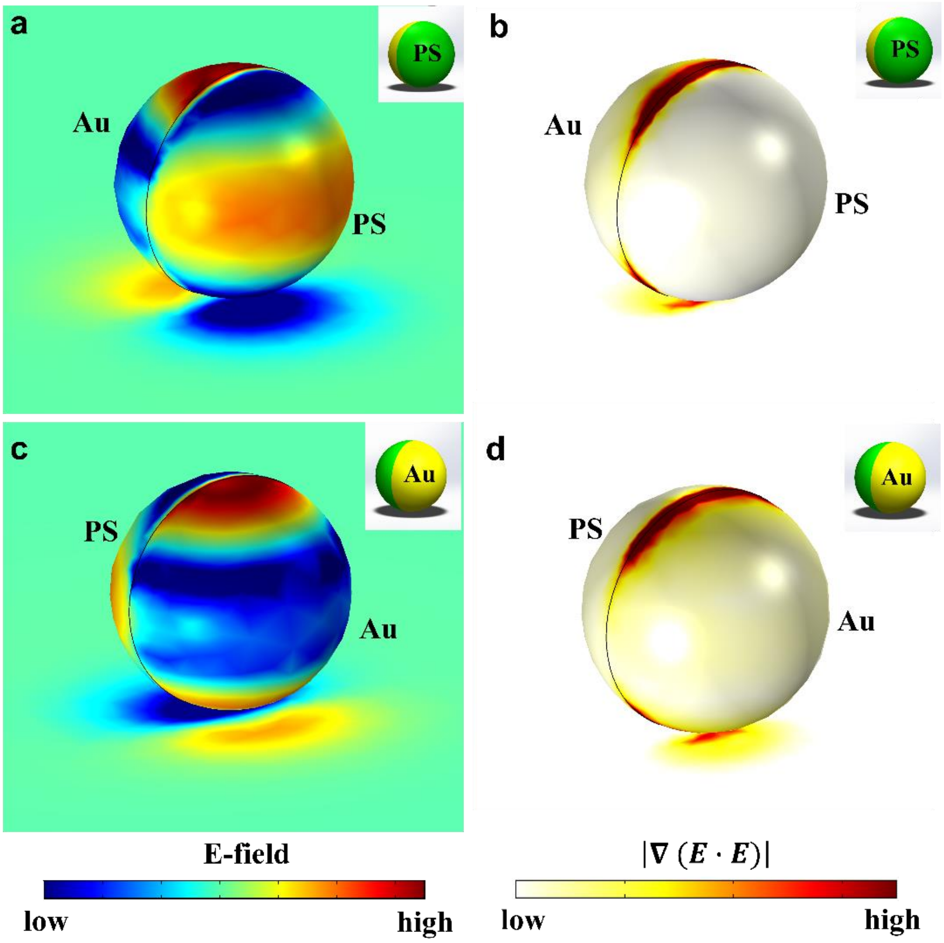

Figure S3. 3D simulation of the electric field norm and its gradient onto the 27µm metallo-dielectric Janus particles. (a, c) electric field norm (b, d) its gradient on the dielectric and metallic surfaces, respectively.

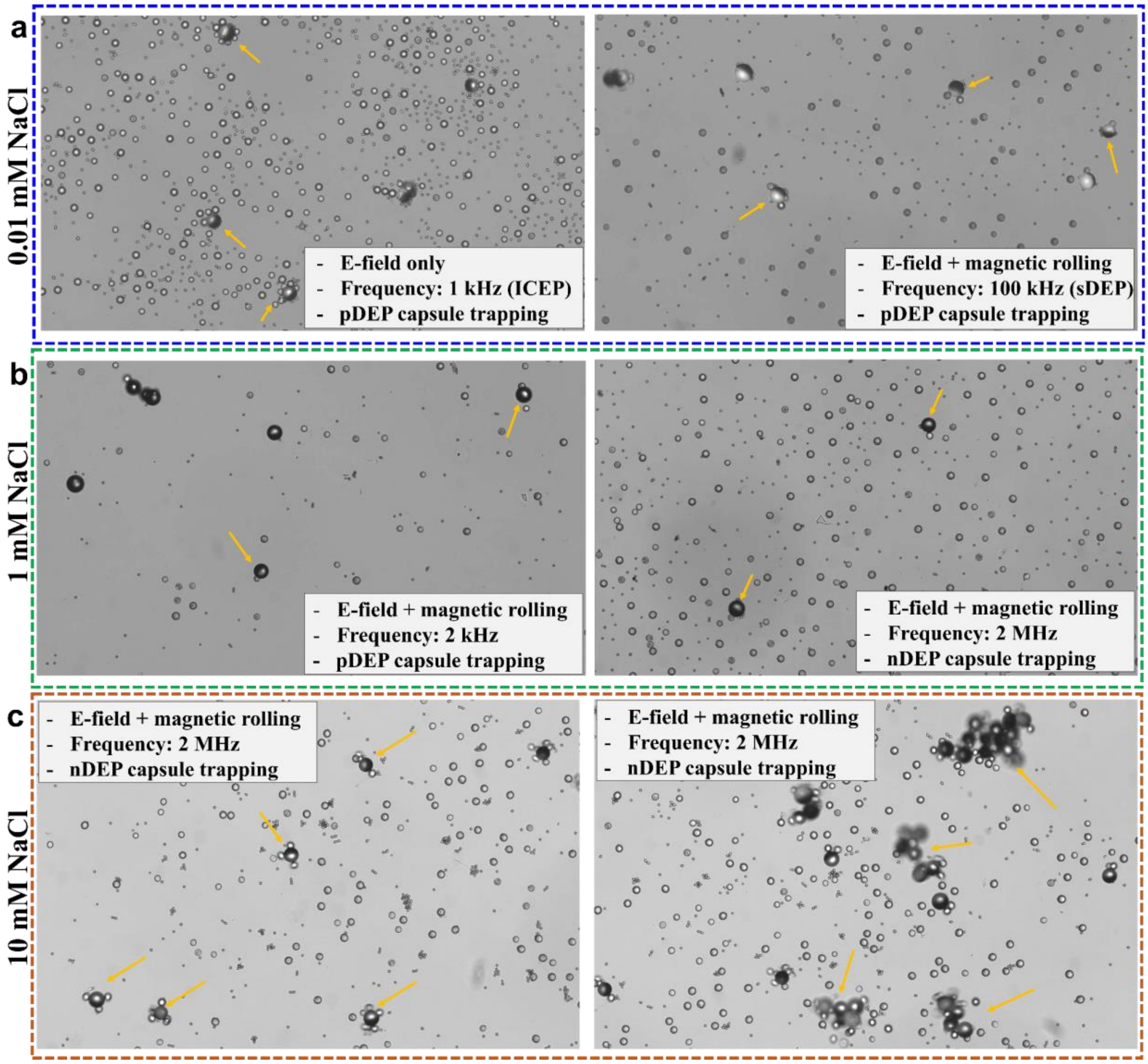

Figure S4. Transport behavior of assembled biohybrid microrobots under varying frequencies of the applied external electric field and different NaCl concentrations using 27µm metallo-dielectric JPs. (a) Comparison of the propulsion and cargo loading capacity using pDEP trapping of capsules between only electric field application with ICEP propulsion mode and a combination of magnetic rolling and electric field application with sDEP propulsion mode wtihin 0.1 mM NaCl. (b) Comparison of pDEP and nDEP capsule trapping under the applied electric field frequencies of 2kHz and 2 MHz, respectively, within 1 mM NaCl. (c) Comparison of individual JPs and clusters of JPs based assembly with capsules within 10mM NaCl.

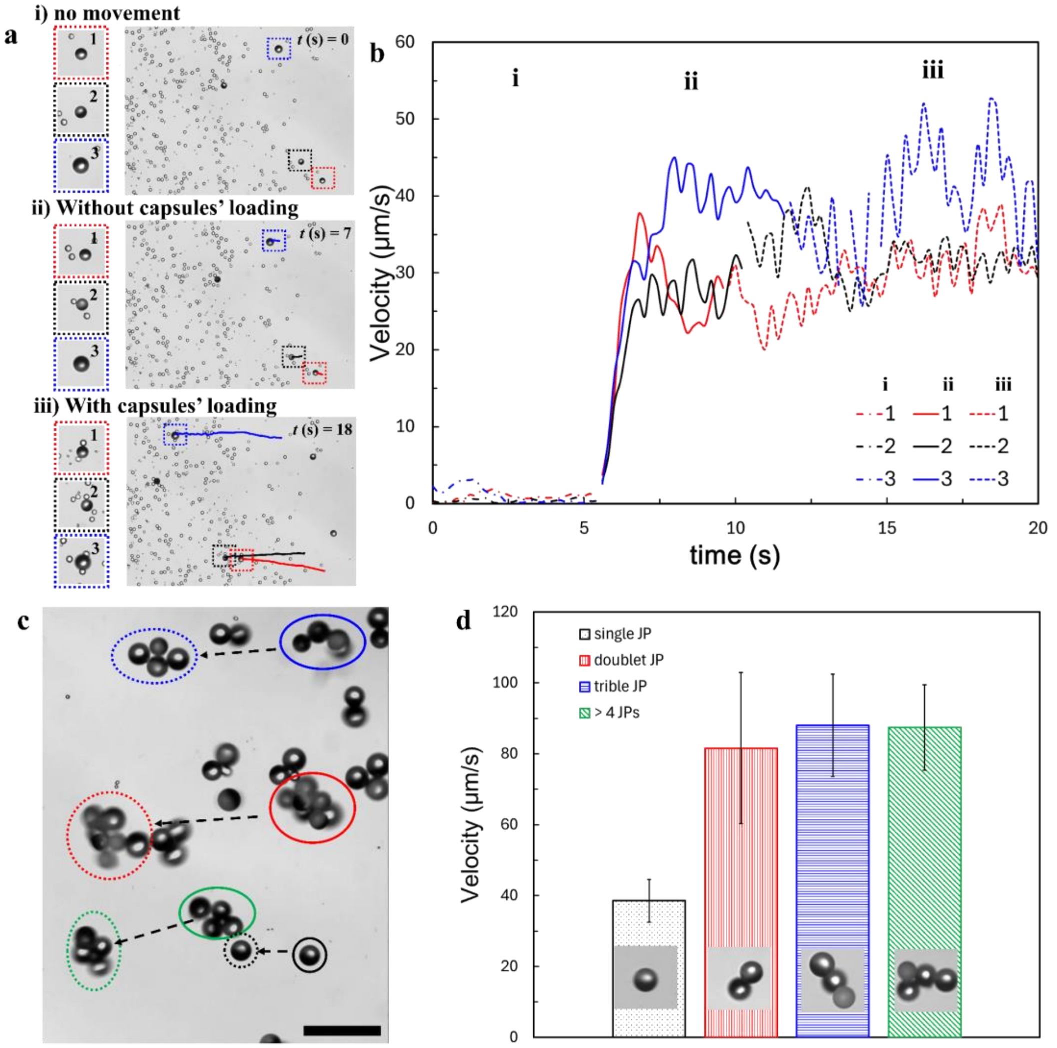

Figure S5. Transport of bare Janus Particles (JPs) and assembled JPs and stinging capsules (i.e. biohybrid microbots (MRs)) under combined magnetic rolling and electric field applications. (a) Representative trajectories of JPs (labeled JP 1, 2, 3) in a 10 mM NaCl solution under three conditions: (i) no field application, (ii) JPs without loaded capsules, and (iii) biohybrid MRs with loaded capsules. Red, black, and blue-dotted rectangles indicate the positions of single JP 1, 2, and 3, respectively. The applied magnetic rolling field was set at 100 rpm, and the electric field was 2 MHz with 15 V_{pp}. (b) Corresponding velocity profiles of single JPs (JP 1, 2, 3) under the same conditions as described in (a). Dash-dot, solid, and dashed lines represent the time intervals for no transport in the absence of applied fields, transport of bare JPs without capsules, and transport of biohybrid MRs formed by assembled JPs and capsules, respectively, under the combined electric and magnetic fields. (c) Superimposed microscopic image showing the propulsion of single JPs (black rectangle) and assemblies of more than four JPs (red, blue, and green rectangles). Black dashed arrows indicate the direction and displacement of each JPs. Solid and dotted rectangles denote the respective initial and final positions of JPs over a 3-second interval. (d) Average linear velocity profiles of JPs in various configurations: single, doublet, triplet, and assemblies of more than four JPs, without capsule loading. Errors were generated by examining at least 4 JPs for each case.

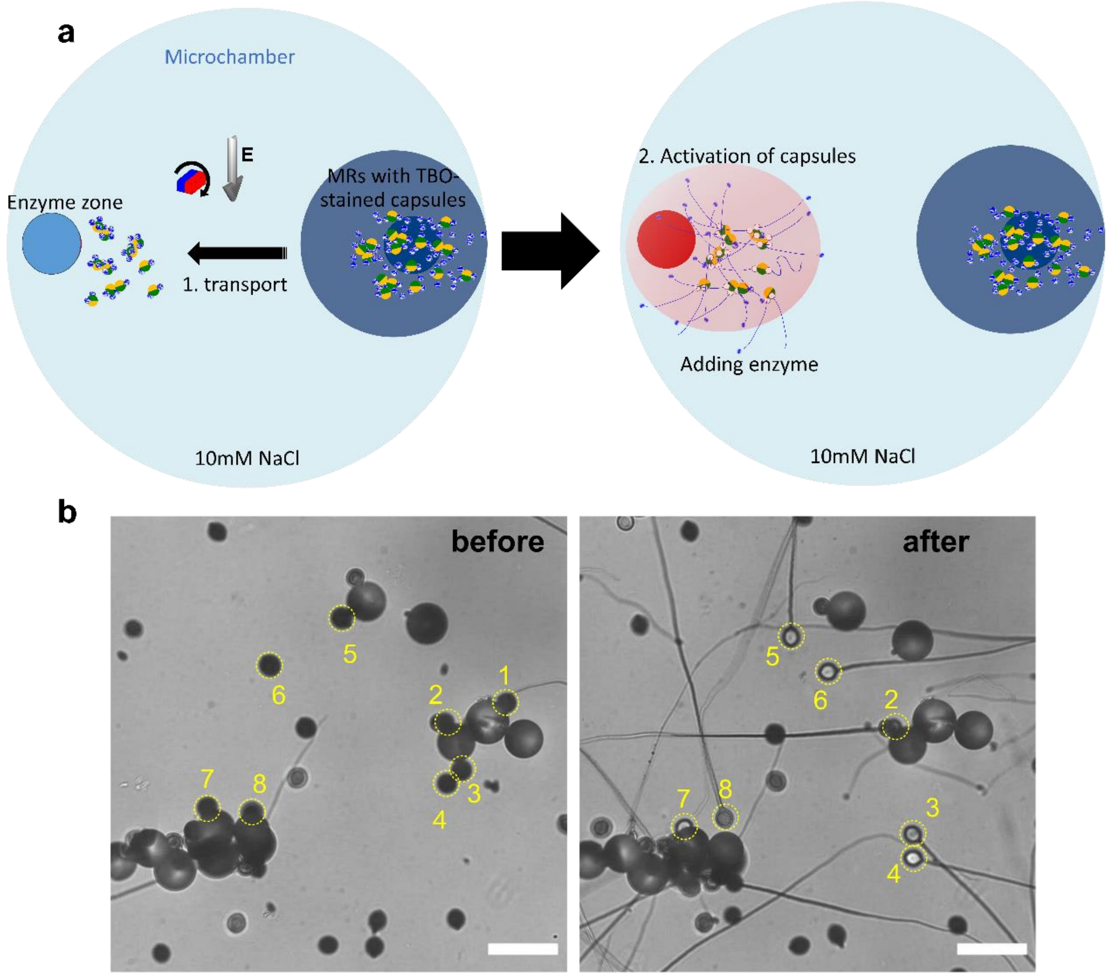

Figure S6. Molecule ejections from biohybrid microrobots by enzyme triggering of the jellyfish capsules. (a) Schematics of the molecule ejection procedure, illustrating (i) the transportation of assembled biohybrid microbots to the left side of the microchamber, and (ii) activation of the loaded capsules by introducing enzyme. (b) Microscopic images depicting biohybrid microbots before and after activation. Yellow dot circles with numbers indicate the marked jellyfish capsules before and after activation, highlighting the release of preloaded Toluidine Blue O molecules from the capsule through their released tubules.

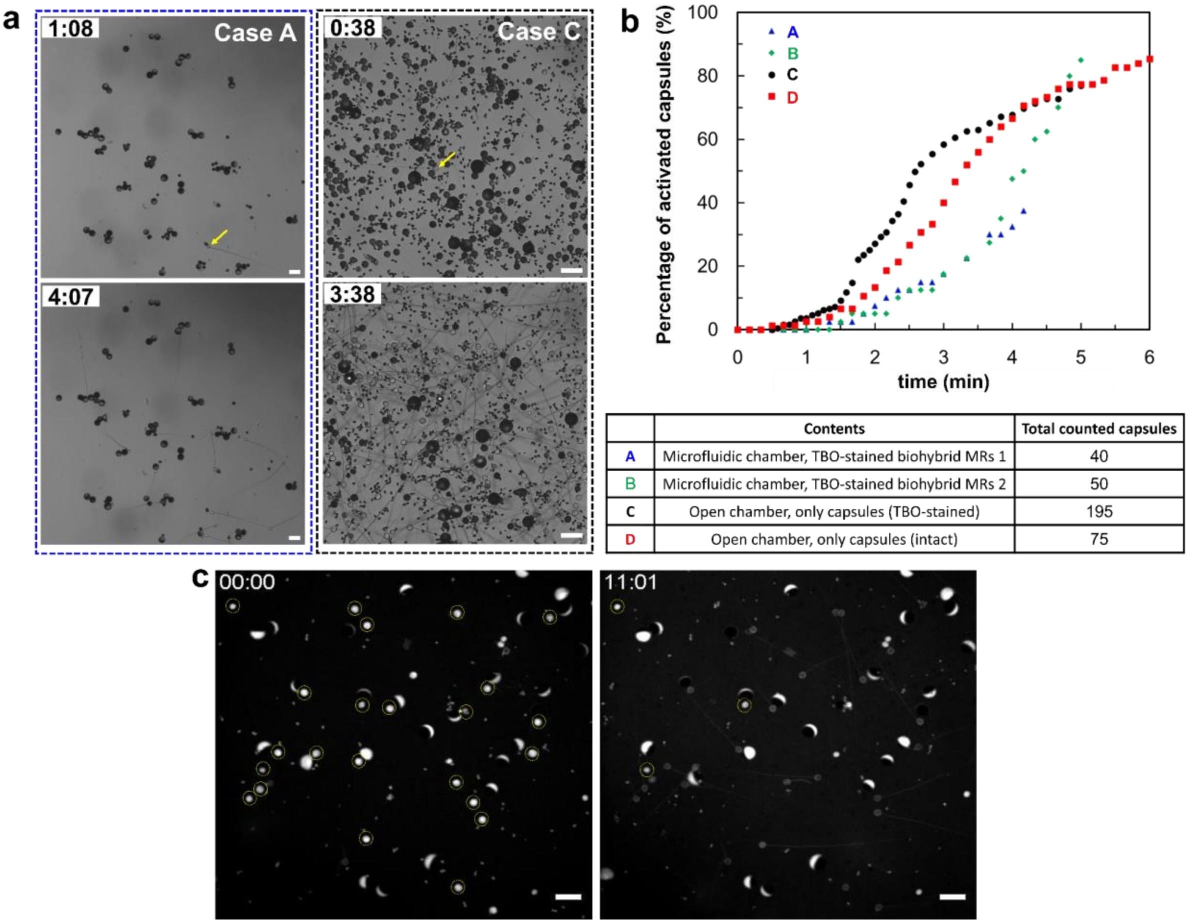

	Contents	Total counted capsules
A	Microfluidic chamber, TBO-stained biohybrid MRs 1	40
B	Microfluidic chamber, TBO-stained biohybrid MRs 2	50
C	Open chamber, only capsules (TBO-stained)	195
D	Open chamber, only capsules (intact)	75

Figure S7. Time evolution of molecule-preloaded capsule activation and ejection. (a) Representative time-lapse microscopic images showing the first tubule activation (t = 68 s and 38 s) after enzyme introduction (t = 0 s) and 3 minutes after first activation (t = 247 s and 218 s) for biohybrid MRs with toluidine blue O (TBO)-preloaded capsules (A, blue dashed line) and TBO-preloaded capsules alone (C, black dashed line), respectively. (b) Corresponding time-evolution graph illustrating the percentage of activated capsules with ejected tubules relative to the total capsules counted before activation across four conditions: A, B – transport of TBO-preloaded biohybrid MRs within a microfluidic chamber (~1 mm from the left inlet); C – TBO-preloaded capsules alone in an open chamber; D – intact capsules (without encapsulated molecules) in an open chamber. (c) Representative time-lapse microscopic images showing capsule activation from biohybrid MRs preloaded with the fluorescent molecule acridine orange hemi(zinc chloride) salt after sufficient enzyme exposure (11 minutes) within a microfluidic chamber.

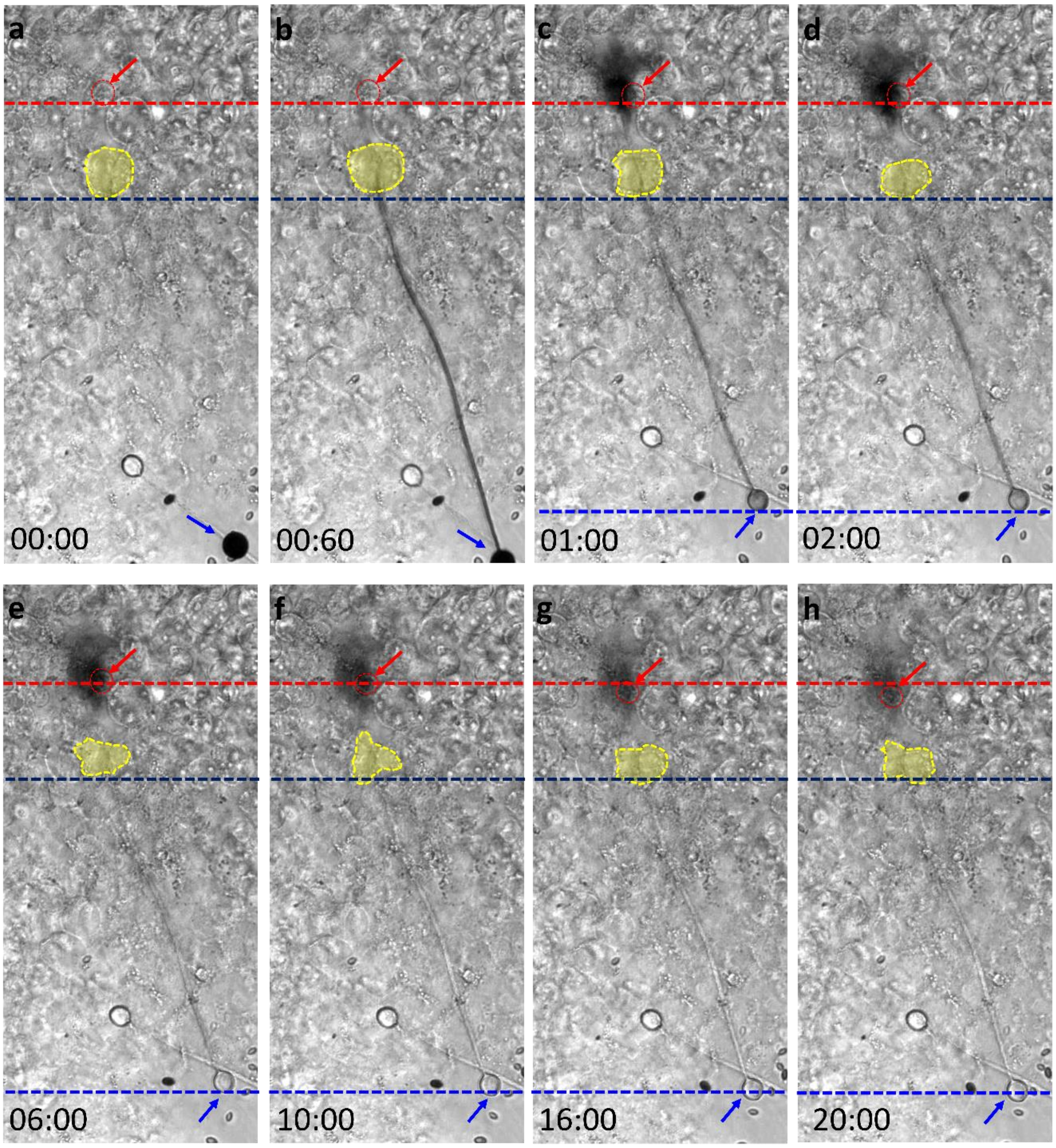

Figure S8. Zoomed-in time-lapse microscopy images illustrating the penetration of the ejected tubule into the tumor spheroid, followed by the injection process (taken from Movie S6, Figure 4c). The red, navy, and blue dotted lines indicate the locations of two representative cells inside the organoid and the capsule body outside the organoid, respectively, demonstrating cellular movement in response to tubule activity. The yellow shape highlights a cell inside the organoid that undergoes deformation due to direct tubule penetration.

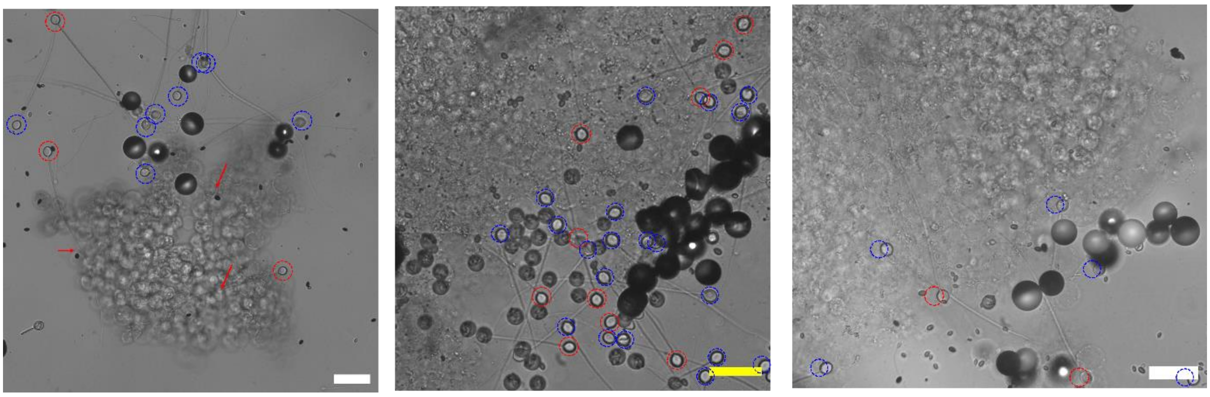

Figure S9. Percentage of tubule penetration into a cancer spheroid by activated capsules. Red dashed circles indicate successful penetration, while blue dashed circles highlight unsuccessful attempts. The spheroid volumes are 0.006, 0.007, and 0.01 mm^3 (left to right).
